# Recurrent chondroblastoma of the talus: A case report and literature review of recurrent lesions in the foot and ankle

**DOI:** 10.1016/j.ijscr.2023.108192

**Published:** 2023-04-14

**Authors:** Olivia Jagiella-Lodise, Timothy McAleese, Mark Curtin, Alan Molloy, James Walsh

**Affiliations:** a Department of Orthopaedics, Beaumont Hospital, Dublin, Ireland; b Department of Orthopaedics, Cappagh National Orthopaedic Hospital, Dublin, Ireland

**Keywords:** Chondroblastoma, Talus, Recurrence, Intralesional curettage, En bloc resection

## Abstract

**Introduction and importance:**

Chondroblastoma is a benign cartilaginous tumour that usually presents in the epiphysis of long bones in patients aged 10–20 years old. Only 4 % of primary chondroblastoma occur in the talus. Recurrence is rare, especially in the foot and ankle and there is no consensus regarding how it is best managed. This unique case and literature review add to a limited evidence base.

**Case presentation:**

A 21-year-old male was referred to our elective orthopaedic clinic with persistent anterior ankle pain exacerbated by weight-bearing. Radiographs and MRI revealed a 2.5 cm non-homogenous mass in the anteromedial talus with expansion of overlying bone consistent with chondroblastoma-ABC. Our patient was initially managed by intralesional curettage and autologous bone grafting but had recurrence 4.5 months postoperatively. Subsequent en bloc resection of the talar neck with talonavicular and calcaneocuboid joint fusion resulted in excellent functional outcomes and disease-free survival at 2 years follow-up.

**Clinical discussion:**

There are few reports discussing treatment options for recurrence in the foot and ankle. Successful treatment of primary and recurrent lesions depends on complete local resection. Repeat curettage or en bloc resection are effective options depending on tumour size and location. Type of bone graft or void filler should be considered on a case-by-case basis. Novel therapies (e.g. phenol instillation) may be beneficial.

**Conclusion:**

This case details successful management of recurrent chondroblastoma with en bloc resection of the talar neck and hindfoot reconstruction. We review the efficacy and outcomes of all previously reported recurrent chondroblastoma in the foot and ankle. We highlight multiple potential treatment options.

## Highlights

• Only 4 % of primary chondroblastoma present in the talus.
• This case describes recurrent chondroblastoma of the talus managed by en bloc resection and joint fusion.
• There is no consensus regarding the management of recurrent lesions in the foot and ankle.
• Recurrence of chondroblastoma is 10–35 %.
• Novel adjuvant therapies have a potential role in preventing chondroblastoma recurrence.

## 1 Introduction

Chondroblastoma (CB) is a rare (1 % of bone tumours), cartilaginous tumour that usually affects the long bone epiphyses in patients aged 10-20 years-old [1]. It is estimated that between 3 %–16 % of chondroblastoma cases involve the foot and ankle, but that only 4 % occur in the talus [2], [3].

CB is most often benign but can metastasize [4]. The typical management of primary chondroblastoma is aggressive curettage and subsequent void filling with bone graft or cement [5]. Recurrence rates are reported between 10 %–35 % although the specific risks for recurrence are unclear [3], [5]. The presence of a secondary aneurysmal bone cyst (ABC) has been previously linked with recurrence but this association remains disputed [6].

The current literature concerning the treatment of recurrent chondroblastoma focuses on long bones. This case is the first to report successful management of recurrence in the talus by en bloc resection and hindfoot reconstruction. Our literature review amalgamates the outcomes of previously reported recurrences of CB in the foot and ankle.

This case report meets SCARE criteria [7].

## 2 Presentation of case

A 21-year-old male was referred to our orthopaedic clinic for persistent anterior, right ankle pain. Twelve months previously, he had rolled his ankle for which he attended physiotherapy for a presumed sprained ankle. However, the pain intensified, prompting a presention to the emergency department and then our clinic. His chief complaint was ankle pain exacerbated by weightbearing.

He also reported intermittent ankle swelling, especially after strenuous exercise. No other joints were affected and his activities of daily living were not limited. He denied systemic symptoms such as weight loss and fatigue. He had no background medical or surgical history, he denied smoking and any family history of cancer.

Clinical exam revealed point tenderness anteriorly. His gait was smooth and symmetrical, he was fully weight-bearing and had full range of motion in his ankle. There was no lymphadenopathy in the groin or popliteal fossa and he was otherwise neurovascularly intact.

**Fig. 1:**
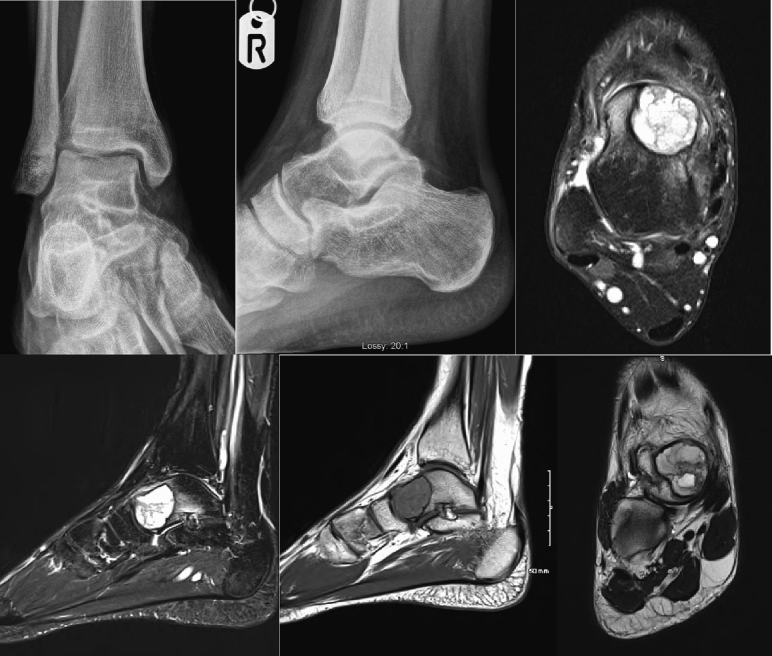
(A, B, C, D, E, F): Pre-operative AP and Lateral Ankle radiograph showed a lucent lesion within the talar neck No periosteal reaction was identified. There was no pathologic fracture. Alignment the ankle mortise was normal. Preoperative MRI demonstrated a 2.5 cm cystic mass in anteromedial aspect of talus with possible slight expansion of the overlying bone. There was extensive surrounding bone oedema in the distal half of the talus, predominantly the neck and subarticular aspect of the talonavicular joint. This was non-homogenous. The ankle joint appeared normal with an appropriate amount of fluid. There was a normal amount of fluid in the small joints of the foot and the ligaments and tendons were all intact.

Radiographs showed a lucent lesion within the talar neck without fracture or periosteal reaction. Overall alignment of the ankle mortise was normal (Fig. 1). MRI reported a 2.5 cm, non-homogenous mass in the anteromedial talus with expansion of the overlying bone. Bone oedema surrounded the talar neck and subarticular aspect of the talonavicular joint (Fig. 1).

**Fig. 2:**
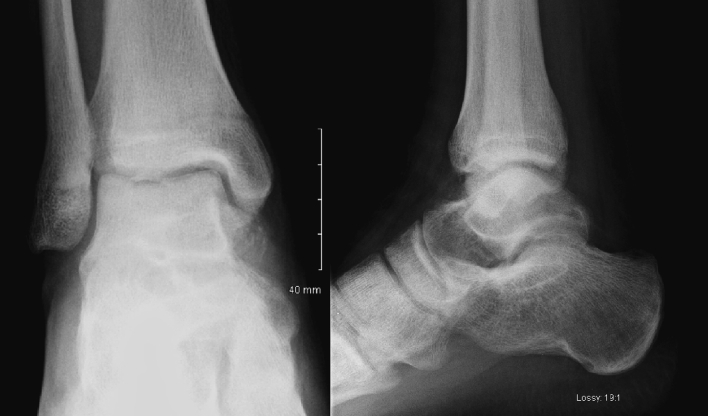
(A, B): Post-operative AP and Lateral Ankle radiographs at 2 weeks. There was a heterogeneous appearance within the anterior process of the talus, in keeping with the recent surgical excision. There was interval development of marked osteopenia, particularly within the mid and hindfoot. There was also a further ill-defined periosteal reaction surrounding the bones of the midfoot.

Over the next 2 months, our patient became gradually unable to weightbear to the point where he required crutches. He subsequently underwent intralesional curettage and bone grafting via a single anterior approach to the talus. A cortical window was used to expose the tumour cavity before an aggressive curettage was performed. The remaining void was filled with autologous iliac crest bone grafting. Our patient reported immediate relief of symptoms. Post-operative radiographs were satisfactory (Fig. 2). He was advised non-weightbearing for 6 weeks post-operatively.

Histological examination of the intraoperative samples revealed mild cytological atypia with moderate numbers of multi-nucleated giant cells and scattered epithelioid cells. A significant cystic component was present which was noted to be more cellular than the usual fibrotic wall of an ABC. Areas of chondroid differentiation and necrosis were present throughout the lesion. Immunohistochemistry demonstrated weak positivity for S100, CD-68 staining histiocytes, and the MIB1 proliferation index was 5–10 %. The mitotic count was 1–2/10HPF. After multidisciplinary discussion the lesion was diagnosed as a benign chondroblastoma with secondary aneurysmal bone cyst formation.

**Fig. 3:**
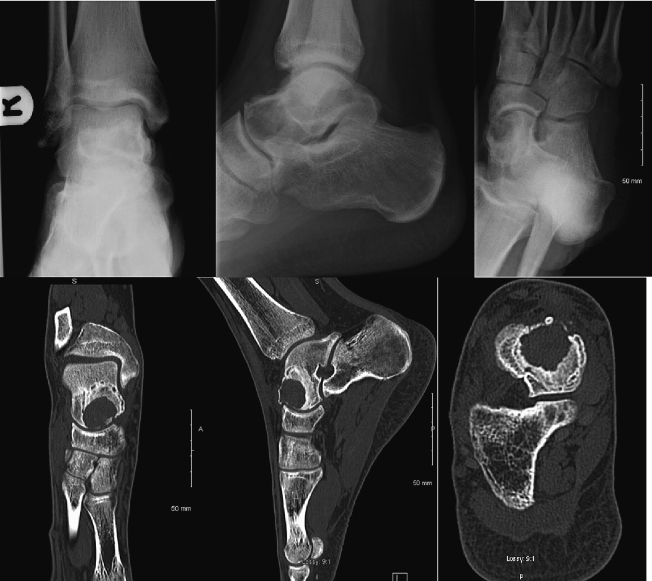
(A, B, C, D, E, F): Ankle radiographs at 4.5 months post initial curettage and grafting. There was a persistent, rounded focal lucency identified within the medial aspect of the head of the talus (2.2 cm). This lesion was non-aggressive in appearance. There was minor bone spur formation at the neck of the talus compatible with chronic anterior ankle impingement. The ankle, the subtalar, calcaneocuboid, and the bones and joints of the mid and forefoot were well preserved. Coronal, Sagittal and Axial CT images showed recurrence of a 2.2 × 2.4 cm heterogeneous cystic lesion in the head of the right talus. There was a thin sclerotic margin and a breach of the overlying cortex anteriorly and superiorly, in keeping with the previous surgical excision. There was surrounding periosteal reaction and soft tissue thickening in the dorsal aspect of the foot. No fracture was identified. Right ankle joint bones were preserved.

**Fig. 4:**
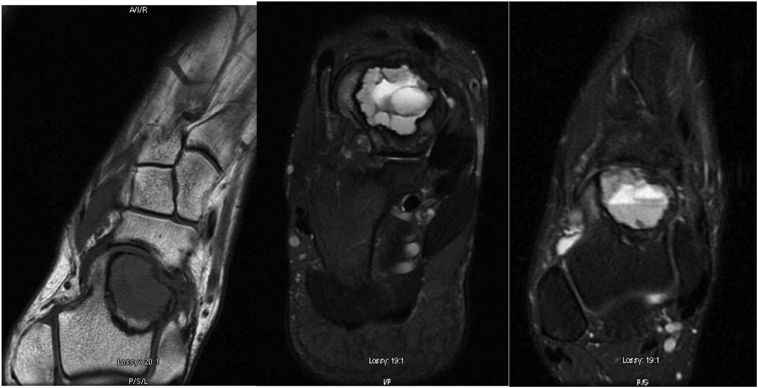
(A, B, C): Coronal T1, Axial T2, Coronal SE MRI images. Post-recurrence MRI showed a multiloculated expansile cystic lesion within the neck and head of the talus measuring 2.6 × 2.3 × 2.1 cm with a well-defined sclerotic margin correlating with the known chondroblastoma-ABC. Minor adjacent osseous oedema was present which had diminished significantly when it was compared with the prior pre-operative MRI. Otherwise, there were no suspicious features or significant new findings apparent.

At routine follow-up, 4.5 months post-operatively, repeat plain radiographs identified a persistent focal lucency within the talus (2.2 cm) (Fig. 3). Chest X-ray was clear of metastatic disease. CT confirmed recurrence of a heterogeneous, cystic lesion in the talus with no associated fracture (Fig. 3). Repeat MRI revealed a multiloculated, expansile, cystic lesion within the neck and head of the talus (2.6 × 2.3 × 2.1 cm) with a well-defined sclerotic margin (Fig. 4). This case was discussed at the orthopaedic multidisciplinary meeting and with the national orthopaedic oncology centre. It was decided the patient should undergo revision surgery with en bloc resection.

**Fig. 5:**
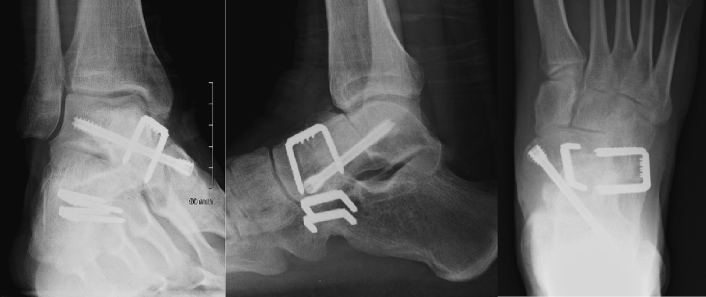
(A, B, C): Radiographs at 6 weeks post en bloc resection of the talar chondroblastoma. This radiograph demonstrates en bloc resection, femoral head allograft reconstruction of the talus and screw/staple fusion of the talonavicular and calcaneocuboid joints.

The revision procedure was performed through dual incisions, a curvilinear dorsolateral approach and a single anterior approach to the talus as before. Anteriorly, the talar neck was resected as far back as the talar dome and the articular surface of the talonavicular joint was excised. The remaining interval was prepared using a birr, drill and curettage before the defect was filled with a block of femoral head allograft. Graft fixation and talonavicular fusion were achieved using a 6.5 mm cannulated compression screw and 18 mm fixation staple. Next the calcaneocuboid joint and sinus tarsi were exposed through a dorsolateral approach. This joint was prepared with a birr and drill before it was fused using two 18 mm fixation staples (Fig. 5).

Two years later, our patient reports excellent functional outcomes, no pain and remains disease-free. His only symptom is mild ankle stiffness after strenuous exercising, which always resolves after stretching and warm showers. He explains he is aware of mildly limited inversion/eversion of his foot although this does not bother him or affect his activities of daily living. His lower limb Musculoskeletal Tumour Society (MSTS) score was 30/30, and American Orthopaedic Foot and Ankle Society (AOFAS) Hindfoot score was 94/100.

## 3 Discussion

Chondroblastoma typically presents as chronic, non-traumatic arthralgia [1]. Non-specific, long-term symptoms often delay the diagnosis of these lesions [4]. Furthermore, chondroblastoma, giant cell tumours and ABCs share similar clinical and radiological findings and can also be present simultaneously, compounding the challenge of accurate diagnosis [8].

Primary foot and ankle involvement is rare, most commonly involving the calcaneus and talus [3]. An osteolytic lesion is seen on radiographs which provokes further imaging. Cross-sectional imaging is referenced to estimate tumour volume, articular surface involvement, soft tissue involvement or for cortical breaches, which are associated with aggressive tumours [9]. Histological evaluation confirms the definitive diagnosis [10]. The intercellular matrix stains positive for S-100 and CD-68 [10]. Recently, the H3F3 K36M mutant antibody has been proven sensitive and specific for chondroblastoma [11]. Secondary ABC is associated with 15 %–32 % of chondroblastoma [12]. This is more common in pedal chondroblastoma and more destructive lesions [12]. It has also previously been associated with recurrence [2], [6] although this has not been found in other case series [5].

### 3.1 Recurrence

Recurrence rates are reported between 10 %–35 % [3], [5], [6]. This has been shown to correlate with curettage margins and ensuring no seeding of tumour cells during the primary intervention [1], [13], [14]. The most high-risk areas for recurrence are near the epiphyseal growth plate and articular surface where aggressive curettage is unfavourable [5]. In the foot and ankle, areas with limited surgical access such as the tarsal bones and their facets are more at risk of incomplete curettage.

Surgery remains the gold standard of CB treatment [4]. Tumour location and volume determine approach for both index and revision operations. Tumour activity indicates whether more aggressive treatment such as en bloc resection or amputation is indicated [15]. The most common surgical intervention is intralesional curettage and void filling. Aggressive curettage with bone grafting, bone substitutes or cementing offers local control of tumour growth with good outcomes [4], [13], [16]. Specific to the foot and ankle, Angelini et al. analysed 40 cases of primary CB and found intralesional curettage and packing was curative in 97.5 % of cases [3]. En bloc resection is the recommended surgical approach for larger or more aggressive lesions, and offers lower risk of recurrence than curettage. However, en bloc resection is not always necessary based on tumour location or volume and can be associated with slightly lower MSTS scores compared to curettage [4], [15].

### 3.2 Management and outcomes

While there are several reports of long bone recurrence, our literature review demonstrates a total of 18 reported cases of recurrent chondroblastoma in the foot and ankle. Including this case, 4 involve the talus. The calcaneus was the most common bone involved. The mean time to recurrence was 20.4 months and patients were predominantly male (Table 1).

**Table 1:** Summary and comparison of all chondroblastoma recurrences in the foot and ankle.

Author	Age	Sex	Location	ABC	Initial surgery	Time to local recurrence	Recurrence treatment	Outcome
Jagiella-Lodise et al. (current)	21	M	Talus	Yes	IC + AU	4.5 months	En bloc resection + AL + fusion	No re-recurrence
Wang et al. (2021)	26	M	Calcaneus	No	IC + AL + BS	5.6 months	IC + AU + Burring	No re-recurrence
Negri et al. (2020)	33	M	Calcaneus	N/A	IC	108 months	a	Lost to follow-up
Outani et al. (2020)	31	M	Calcaneus	N/A	IC	39 months	IC + BS	No re-recurrence
Outani et al. (2020)	22	M	Calcaneus	N/A	IC	18 months	IC + BS	No re-recurrence
Angelini et al. (2018)	20	M	1st toe MTPJ	N/A	IC + grafting	24 months	En bloc resection + MTPJ arthrodesis	No re-recurrence
Konishi et al. (2017)	a	a	Calcaneus	N/A	IC	a	a	a
Dutt et al. (2015)	21	M	Calcaneus	No	IC + AL	15 months	IC + BC + cryosurgery + burring	Lost to follow-up
Fukunaga et al. (2010)	49	F	Multiple tarsal bones	N/A	Radiotherapy	a	a	AWD 7 years
Lin et al. (2005)	27	M	Talus	N/A	IC + AU	19 months	IC + AU	No re-recurrence
Suneja et al. (2005)	13	M	Talus	No	IC	9 months	BKA (extensive hindfoot recurrence)	No re-recurrence
Suneja et al. (2005)	12	M	Calcaneus	No	IC	4 months	BKA	Metastases at 6/12. Died at 4.5 years
Accadbled et al. (2001)	14	M	Talus	Yes	IC + AL	14 months	Wide resection and fibular graft	No re-recurrence
Dahlin et al. (1972)	a	a	Distal tibia	N/A	IC + grafting	9 months	Resection and Arthrodesis	Good result at 45 months
Dahlin et al. (1972)	a	a	Distal tibia	N/A	IC	22 months	IC + grafting	Good result at 6 years
Dahlin et al. (1972)	a	M	Metatarsal	N/A	IC	17 months	Metatarsal resection	Not specified
Schajowicz et al. (1970)	32	F	Metatarsal	N/A	IC + BC	5 months	Metatarsal Resection	DWD
Schajowicz et al. (1970)	33	M	Calcaneus	N/A	IC + BC	14 months	Revision IC x2	No re-recurrence after second IC

ABC: Aneurysmal Bone Cyst, IC: Intralesional curettage, AU: Autograft, AL: Allograft, BS: Bone substitute, BC: Bone cement, BKA: Below Knee Amputation, AWD: Alive with disease. DWD: Died with disease.

a Information not specified in paper.

There is no current consensus on how to treat these lesions. Treatment modalities for recurrence included revision intralesional curettage, wide local/en bloc resection and amputation. Our literature review suggests a trend towards revision curettage although each case was highly individual. Seven patients were treated in this manner with variations in void-filling technique. Six patients had no re-recurrence and one was lost to follow-up. Recurrent chondroblastoma is thought to be more aggressive than primary lesions and should be treated with a high degree of vigilance [4]. This specifically applies to the foot and ankle as three of the patients in our review had metastatic or persistent disease after re-operation; one died of metastasis, one died with disease, and one survived with disease (Table 1).

The effectiveness of revision curettage have been demonstrated in small case series of long bone recurrence although limited published data exists on chondroblastoma recurrence in general. Suneja et al. reported results of seven local recurrences, five in long bones and two in the foot and ankle. The mean time to recurrence was 10 months. Four patients were managed with revision intralesional curettage, one patient with endoprosthetic replacement of the humerus and both foot and ankle recurrences were managed by amputation. Three patients were successfully managed by revision curettage, but one patient underwent two further failed curettages before resection and endoprosthesis [5]. Xu et al. described the effective management of four patients with revision curettage and adjuvant therapies for long bone recurrence [4]. Ozer et al. analysed five long bone recurrences and found that revision curettage and bone cementing was effective in three cases [13].

### 3.3 Void Filling and adjuvant therapies

Revision surgery often involves more aggressive curettage or en bloc resection with wider margins and the need for subsequent reconstruction [14], [17]. Our case involved the talus, prompting major concern for structural integrity of the ankle. Treatment had to address the talus' limited vascular supply, load-bearing status, and importance in foot-ankle mobility. Typically void filling is performed with bone graft or cement for structural support and has the proposed benefit of reducing recurrence [2].

Autograft, allograft, and demineralised bone substitutes have all been show to be effective in small foot and ankle lesions [4], [5], [14]. The main theoretical concern that graft use addresses in the talus is the risk of atrophy or necrosis due to poor blood supply.

While polymethylmethacrylate cement is widely used with good outcomes, it has been associated with growth arrest in immature skeletons [14]. Conversely, benign orthopaedic tumour recurrence can be higher in bone graft compared to cement filler [18]. Other studies show no difference in outcomes based on filling material [17].

Extensive curettage adjacent to articular surfaces is associated with joint degeneration [19]. Adjuvant therapies (e.g. phenol instillation and cryotherapy) have a potential role in recurrence although the majority of current evidence relates to their use in primary CB and other lesions near the physis and articular surface [17].

Recently, denosumab treatment for chondroblastoma has been suggested due to CB's similar profile to giant cell tumours, including RANK L expression and how it responds to this human monoclonal antibody [20].

## 4 Conclusion

Chondroblastoma is a benign lesion with recurrence potential. There remains no consensus regarding most appropriate treatment. Our case report describes successful management of rare, recurrent chondroblastoma of the talus with en bloc resection and hindfoot reconstruction. Our literature review describes treatment modalities for recurrent disease.

## Consent

Written informed consent was obtained from the patient for publication of this case report. Evidence of this can be provided to the Editor-in-Chief of this journal upon request.

## Ethical approval

Ethical approval is exempt/waived by the authors institution.

## Funding

N/A.

## Author contribution

Conceptualization: Alan Molloy and James Walsh

Data curation: Olivia Jagiella-Lodise, Timothy McAleese, Mark Curtin, Alan Molloy, and James Walsh

Supervision: Timothy McAleese, Alan Molloy, and James Walsh

Writing:

Original Draft: Olivia Jagiella-Lodise

Review & Editing: Olivia Jagiella-Lodise and Timothy McAleese

## Guarantor

James Walsh.

## Research registration number

Not applicable.

## Declaration of competing interest

N/A.
